# “Everyone just keeps their eyes closed and their fingers crossed”: Sexual health communication among black parents and children in Nova Scotia, Canada

**DOI:** 10.1186/1475-9276-12-55

**Published:** 2013-07-22

**Authors:** Antoinette N Davis, Jacqueline C Gahagan, Clemon George

**Affiliations:** 1 Gender and Health Promotion Studies Unit, Health Promotion Division, School of Health and Human Performance, Dalhousie University, Halifax, NS, Canada; 2 CIHR New Investigator in the area of HIV/AIDS, Faculty of Health Sciences, University of Ontario Institute of Technology, Oshawa, ON, Canada

**Keywords:** Sexual health, Black youth, Parents, Qualitative research, Nova Scotia, Communication

## Abstract

**Background:**

Black Canadian youth remain disproportionally affected by an array of social and health issues, including sexually transmitted infections. While research exists in support of the involvement of parents as a key means to prevent or modify harmful behaviours among youth, less is known about how parent–child communication can serve as a prevention intervention strategy within Black families in Canada. This study explores sexual health communication between Black parents and youth in Nova Scotia and identifies facilitators, obstacles and issues that families face in dialoguing about sexual health.

**Methods:**

Focus groups and in-depth interview sessions were held with a diverse sample of parents of Black youth, health and education professionals, and Black youth in Nova Scotia, as part of a larger study aimed at exploring parent–child communication on sexual health and HIV. The research team worked in partnership with and received feedback from key informants and a community advisory committee throughout the various stages of this study. All sessions were audio-taped with permission and thematic analysis was carried out on the verbatim transcripts.

**Results:**

Six key themes emerged from the data analysis in relation to parent–child communication within Black families in Nova Scotia: 1. the gendered nature of [sexual] health communication; 2. fear and uncertainty as obstacles; 3. open and honest dialogue from an early age as a facilitator; 4. media as both a catalyst and a barrier; 5. peers as a catalyst; and 6. time constraints as an obstacle.

**Conclusions:**

The findings of this study reveal that parent–child communication regarding sexual health promotion within Black families in Nova Scotia remains varied and is heavily affected by a myriad of intersecting determinants of health faced by Black youth and their parents. Health promotion interventions aimed at fostering and supporting parent–child communication on sexual health must simultaneously target both parents and youth and further, such efforts must engage a high level of cultural competency in order to better meet the needs of this population.

## Introduction

Parent–child communication, defined as the exchange of verbal or non-verbal expression of ideas and feelings between a child and a parent or guardian, can have a significant impact on children’s health and wellbeing. Positive communication between parents and adolescents on issues of sexuality and sexual health has increasingly been recognized as an important determinant in the development of healthy sexuality and sexual behaviour among youth in North America [1]. Studies have shown that open, confident, responsive, and consistent parental communication can lead to improvements in the level of consistent use of condoms and contraceptives [2-7], an increase in communication about sexuality between youth and their sexual partners [2,8-10], and a decrease in risk-taking behaviours that place youth at increased risk of acquiring sexually transmitted infections [11-14]. Although Black North American youth have been identified as a marginalized population at heightened risk for sexually transmitted infections (STIs) and HIV [15,16], the role of parental communication in reducing or preventing risk-taking behaviours among this population remains poorly understood and under-researched, including in the Canadian context in Canada.

With particular reference to Black youth in Canada, they account for 7.8% and 11.9% of the cumulative AIDS cases among youth aged 15–19 and 20–29 respectively [16]. Early sexual debut, as well as negative peer influences and the portrayal of Black sexuality in popular culture, have been identified in a number of US-based studies as factors that have contributed to poor sexual health outcomes among Black youth [17-20]. In addition, studies have noted that many sexual and reproductive health issues and concerns begin as a consequence of a variety of determinants of health including structural inequities such as poverty, social and physical isolation, a lack of education and support, marginalization [21,22] and racism. This has been found to be particularly true for Black Canadian youth. For Black youth and their parents, the reality of underemployment, inadequate access to appropriate housing, and inequities in the provision of health and support services needed for maintaining a good quality of life all serve to increase vulnerability to HIV and STIs [23].

Few sexual health interventions directed at or by Black youth exist in Canada and even fewer exist in the Atlantic region provinces such as Nova Scotia, home to the country’s most historic Black communities. The lack of such interventions also exacerbates the susceptibility of Black youth to poor sexual health outcomes, including a variety of sexually transmitted infections and unintended pregnancies. In the absence of such targeted interventions, ‘sex positive’ parent–child communication on sex, sexuality, and sexual health as a health promotion intervention is especially important [24]. Given these issues, our study specifically explored sexual health communication between Black parents and youth in Nova Scotia in an effort to identify facilitators, obstacles, and issues that families face in dialoguing about sexual health.

## Methods

This two-phased qualitative study used both in-depth interviews and focus group sessions to explore communication on sexual health between Black youth and parents in Nova Scotia, Canada. Inclusion criteria required that participants be 16 years of age or older; a Black youth, a parent or guardian of a Black youth, or an individual with experience working with Black youth; and must have lived in Nova Scotia for at least one year. The study was part of a larger research project on parent–child communication on sexual health and HIV within Black communities in Ontario and Nova Scotia. Ethics permission was granted by Dalhousie University’s Research Ethics Board.

The study was guided by Critical Race Theory (CRT), as this theoretical approach helps to illustrate how issues of race function in relation to Black families’ response to sexual health [25-29]. Racial stratification and social exclusion continue to further structural inequities in health through, for example, the development of health and social policies that produce systems that impact on health outcomes of Black populations in Nova Scotia. Specifically, health and social policies that are meant to support Black people sometimes produce divisions between indigenous Black Nova Scotians and more recent immigrants. “Indigenous Black Nova Scotians” denotes the group of Black Nova Scotians whose families have lived in Nova Scotia for several generations and can trace their ancestry back to the Black Loyalists, who landed in Nova Scotia in the late-18th century during the Loyalist migration [30,31], and the escaped slaves who arrived in the early 19th century [32]. As some Black Nova Scotians may believe that there is a need to align themselves with social norms, they may discontinue their own cultural norms and practices regarding health-related issues, including HIV and STIs. As such our interpretation of the study results is filtered through the lenses of critical race theory and the determinants of health.

In the first phase of this study, a community advisory committee was established and in-depth interviews were conducted with eight key informants. These interviews were used to elucidate the concerns and priorities of Black youth and parents, and informed the development of the semi-structured focus group interview guide used in phase two. The key informants included teachers, school support workers, youth group workers, and community-based employees whose professions brought them into regular contact with Black youth and their parents. Their involvement in the education system and various community organizations provided insight into their experiences and their perceptions of what they believed were the primary health and social issues of concern among Black youth and parents. The data from phase one revealed the following health-related inequities: systemic and institutional racism as an ongoing problem; concern about the negative influence of peer pressure on the actions of youth; difficulty with open communication between Black parents and youth, especially when discussing topics of a sexual nature; lack of culturally-specific sexual health education within the school system, and gender-based differences in parenting styles.

A total of six focus group sessions with 26 participants were held during the second phase of the study in February and March 2012. Focus group sessions were 45 minutes to 2 hours in duration and were organized along the following lines: a. Black parents who had children between the age of 16 and 24 (n = 6); b. parents who were in a biracial relationship and had children between the age of 16 and 24 (n = 4); c. male youth between the age of 16 and 20 (n = 3); d. female youth between the age of 16 and 20 (n = 4); e. older youth between the age of 18 and 24 (n = 3); and f. education and health professionals with in-depth knowledge of, and interaction with, Black youth in Nova Scotia (n = 6). The majority of those who took part in the study were indigenous Black Nova Scotians. Participant characteristics are shown in Table 1.

**Table 1:** Demographic characteristics of focus group participants

**Groups**	**Parents**		**Youth**			**Professionals**	
	**Black**	**Biracial**	**Female**	**Male**	**Older**	**Health/Education**	**Total**
	**(n = 6)**	**(n = 4)**	**(n = 4)**	**(n = 3)**	**(n = 3)**	**(n = 6)**	**(N = 26)**
**Age, y**							
16–19	-	-	3	3	-	-	6
20–29	-	-	1	-	3	-	4
30–39	1	-	-	-	-	2	3
40–49	4	4	-	-	-	1	9
50–59	1	-	-	-	-	1	2
60–69	-	-	-			2	2
**Ethnicity**							
Black	6	3	4	2	3	6	24
Caucasian	-	1	-	-	-	-	1
Biracial	-	-	-	1	-	-	1
**Sex**							
Male	1	1	-	3	1	4	10
Female	5	3	4	-	2	2	16
**Education**							
<High School	-	-	2	3	-	-	5
High School	2	3	1	-	-	-	6
Some University	-	1	-	-	2	-	3
College	2	-	-	-	-	-	2
Bachelors	1	-	1	-	1	1	4
Masters	1	-	-	-	-	5	6

The recruitment of focus group participants was carried out by circulating flyers, through word of mouth, and by engaging education professionals and community-based partners with connections to the Black community. Participants received a $15 honorarium to help compensate them for their time away from their other duties and to help cover travel expenses. All six focus group sessions were consistent in terms of topics covered and questions asked. For instance, each focus group discussion began by asking, “What are the most important issues facing young Black men and women in Nova Scotia?” in order to ascertain principal issues of concerns from the participant’s vantage point. Table 2 contains a selection of the material that was discussed.

**Table 2:** Selection of focus group questions

1	What are the most important issues facing young Black men and women in NS? What makes you think so?
2	Do Black parents talk about dating? What things do they say?
3	How does the question/conversation about HIV prevention or sexual health come around? (What precipitates such conversation?)
4	Who talks [to you] about sexual health (or sexuality, including homosexuality)? Mother/Father (male or female caregiver)?
5	Where have you gained most of your knowledge on sex (or sexuality more generally)? HIV prevention?
6	How important are friends or peers regarding sexual health and/or HIV prevention? And parents?

With permission from the respondents, interview sessions were audio recorded and later transcribed verbatim by a trained transcriptionist. A research team member analyzed transcripts using an inductive analysis technique, which allowed relevant themes and categories to emerge from the focus group discussions. First, following each focus group session, the transcript was examined in its entirety and handwritten notes were made about the ideas that the session elicited on the issues of interest. Following completion of all focus group sessions, the transcripts were re-examined to reveal similarities and differences in the information shared and loose themes were created based on review and repetition of the data within and across the focus groups. Afterwards, an additional research team member reviewed transcripts and the themes were revised accordingly. Finally, excerpts from focus group discussions which best illustrated these themes were selected.

## Results

Six key themes emerged from the data in relation to the facilitators, obstacles and issues that Black families face in dialoguing about sexual health. These themes included the gendered nature of [sexual] health communication; fear and uncertainty as obstacles; open and honest dialogue from an early age as a facilitator; media as a catalyst and a barrier; peers as a catalyst; and time constraints as an obstacle. Each theme will be discussed in detail in the following section.

### Theme 1: The gendered nature of [sexual] health communication

In keeping with the broader health promotion and determinants of health literature concerned with gender-based issues and sexual health, we found in our study that, women, and mothers in particular, identified themselves as the ones who discussed sexual health-related issues with their children in the home [33]. One female parent noted, “Fathers have a tendency not to talk about too many things. It’s more the mother. Same thing when I was growing up.” Similarly, a male participant in a subsequent focus group session said, “[My wife] has got to be the one that does all that talking cause I’m a guy…I’m from the old school.” However, discussions with youth revealed that for male youth, fathers, brothers, or in the case of the absence of both, sisters, delivered messages about sex and sexual health. While female youth cited as their mothers as their primary source of information, other sources such as school nurses or other relatives were sometimes mentioned as preferred sources of information.

A number of participants expressed that fathers were more likely to discuss issues such as the value of healthy relationships with both sons and daughters:

*I’ve got to keep telling him you know you’ve got to have respect for women and not just tell him but show him as well, and do all of those things, but you know not all kids have that.* (Health and Education Professionals Focus Group)

*[My daughters are] his stepchildren so he took on the role of talking to them about men in general, and relationships. Stay away from the whole sexual, that’s me…he would buy them roses at Valentine’s every year, just do those little extra things to make them feel like you know lovely young women and then he would say ‘Now when you meet somebody, this is the way they’re supposed to treat you. They’re supposed to be treating you with respect, treating you like a lady’. He tried to teach them that so that they don’t think they need those bad boys to feel that.* (Mother, Biracial Parents Focus Group)

While a number of the participants, primarily the mothers, reported having had open and frank discussions about sex and dating with their children, others participants indicated that this had not occurred in their family. One mother revealed that her son was reluctant to talk to her about such issues and another mother stated that her daughter tended to talk more with her father about serious issues so that she only found out about them afterwards. One mother explained that she tried to curtail her child’s sexual behaviour by forbidding him to date and ensured that he was always in group situations, though she admitted, “I know that things happened that I didn’t know about cause I heard stories later”. One mother felt that sex was a topic of conversation that was best left in the hands of “somebody other…somebody trusted [like] your pastor.”

Sexual health discussions between mothers and their teenagers typically centered on contraceptive methods to avoid pregnancy and seldom, if ever, focused on STIs, which, both parents and youth admitted, were seen as much less of a pressing reality compared to pregnancy. Male youth reported being told by their mothers not to get a girl pregnant, without being given more specific sexual health or STI prevention information while one female youth remarked that her mother didn’t talk to her about such matters because her mother didn’t think that she was sexually active. Some youth viewed discussions with their mother as uncomfortable either because of the topic or because conversations with their mothers were difficult to initiate:

*When we do talk about it, we kind of have to keep it like a joking topic because it’s kind of weird if my mother and me, her son, talking about sex and all that. I just, some things where I think parents shouldn’t touch down on* (Male, Youth Focus Group)

*I would like to talk to her sometimes but… she doesn’t make it easy enough for me to be like ‘mom this is what happened’ or ‘mom this is what I want to talk to you about’. It’s like top secret* (Female, Youth Focus Group)

However, one older female youth admitted that although she resisted, her mother, “talked to me about everything so I was aware of a lot of things, even before school taught me… I have her knowledge, I have school and I have the course that I took in university”.

Teachers were viewed by a number of parents, and almost all of the health and education professionals, as trusted figures who were approached by youth, males in particular, with sexual health-related questions. As one participant remarked,

*Our children, or students, would rather go to another person like yourself, guidance teacher, or someone [else] instead of talking to their parent. They feel they can talk to that person instead of to the parent themselves, so you know it’s good that someone is there for them, because it’s too much with everything that’s going on, you know* (Mother, Black Parents Focus Group)

*It depends on the teacher, you know. Like you have to build up a relationship with the student…you know [a male teacher] could not broach that conversation with a female [student], but he would have to have a relationship with a male [student]…so that [the student] knows who to talk [to]* (Male, Education and Health Professionals Focus Group)

### Theme 2: Fear and uncertainty as obstacles

Fear and uncertainty were often the feelings expressed by participants when it came to discussions of sex, sexuality and sexual health. For female youth, the fear and uncertainty that came with not knowing how their parents might react to their questions created a barrier to dialogue on issues of sexuality. Fear of upsetting their parents or casting doubts in their parents’ minds about the appropriateness of their behaviour also stifled communication:

*My brother had a child with his girlfriend, well wife now, and they were young, so it’s a big deal for [my mother] for me not to have sex. So [if she knew] that I am having sex, she would lose her mind. Yah, so I just keep that on the down low* (Female, Youth Focus Group)

For many parents, the fear and uncertainty that they felt when broaching topics of sex and sexuality had deep roots in the way that such discussions had been handled when they were young. Almost all of the focus group respondents from the Black, Biracial, and Health and Education Professionals Focus Groups recalled how sex and sexuality had been shrouded in mystery and silence during their own adolescent years. Discussion of sex or sexual health was virtually non- existent in schools, the home or the church, with the exception of the promotion of abstinence in some households. Parents shared how their knowledge of sex and sexual health promotion occurred after marriage or through trial and error and as a consequence, sometimes resulted in unintended pregnancies for themselves, their partners or their siblings. Many of the parents who had grown up without receiving guidance on sexual health from their own parents expressed both a desire to teach their children what they had not been taught (in order to save them from the same pitfalls) and a sense of uncertainty about how best to go about the task of educating their children on a topic that they had received such little advice. What should they say? When should they say it? How should they phrase it so their kids would listen? How would they know if what they said was the correct thing to say? As one participant remarked:

*I have younger children and I’m thinking to myself, like my oldest is only six, and I’m thinking ‘yah, who’s really going to have those conversations?’ Am I really going to have that conversation with my daughter, and what is it that I say to her. You’re always second guessing yourself because, well, if no one taught you how to do it, then how do you know what you’re doing is correct?* (Male, Health and Education Professionals Focus Group)

Another participant from the same focus group asked, “How can you impart your knowledge, if you don’t have any knowledge?” An additional participant admitted, “I don’t know if I want to provoke questions” while a fellow participant summarized Black parent–child communication on sex and sexual health as follows:

*I think when it comes to like sexual health and stuff like that…[for] a lot of parents it’s don’t ask, don’t tell cause they’re kind of afraid [of] the answer that your kid is going to give as to what they’re doing or your kid’s afraid to be honest about what it is they are doing, and everyone just keeps their eyes closed and their fingers crossed hoping that nothing bad is going to happen* (Male, Health and Education Professionals Focus Group)

Although a handful of parents discussed how they had taken condoms away from their children, it remained unclear whether these actions were governed by fear of how the possession of condoms might influence their children’s decision to engage in sex or whether these actions were meant to delay discussion of sex or even communicate their disapproval of the idea of their children engaging in sexual activities:

*Well the guidance counselor that’s at my daughter’s school they gave her condoms. I said, ‘Where did you get those from?’ She said, ‘Mr. So and So gave out condoms’. I said, ‘OK’ then I took them. ‘I’ll hold on to it for you’. I don’t know if I still got them* (Mother, Black Parents Focus Group)

*I go through her bag every night, and I saw condoms and I said, ‘What are these’. She said, ‘Oh we got them in our class today’. I said ‘OK. I’ll take them. I know you’re not using them’* (Mother, Black Parents Focus Group)

*I was at the Gay Pride parade [with my daughter] and they were throwing [condoms] and I got home and my husband says [to my daughter], ‘What are you doing with condoms?’ [My daughter said] ‘They threw them out to people on the street’, [and] I’m like, ‘Oh give me them’. She got the glow in the dark ones and my husband says, ‘What are you doing with condoms?’* (Mother, Black Parents Focus Group)

### Theme 3: Open and honest dialogue from an early age as a facilitator

Many of the parents who had established good communication insisted on the importance of laying the foundation for discussions about sexual health by speaking honestly and openly with their children about their bodies and their sexuality from an early age using age appropriate language. “Right from day one…I was really out with [my son] speaking about sex…I would get down [to] the nitty-gritty,” a parent from the biracial parents focus group asserted. Similarly, two other parents stated:

*Like I said it starts from very young, if you have those conversations with your kids when they’re young and they trust that you’re the reliable source, I mean they’re going to get it from all other ways anyway, but they’re still going to want us somehow if they’re not sure* (Mother, Biracial Parents Focus Group)

*Don’t wait until they get older and already made up their own perceptions of things and then you try to talk to them. I don’t think that’s a good idea. I think parents really lack when they do that* (Mother, Biracial Parents Focus Group)

Parents who had initiated early discussion of sexuality were prompted to do so by a number of factors. For example, for one parent, the idea that her children were being exposed to material of a more sexually explicit nature at a younger age through the school’s sexual health courses reinforced the need to create a dialogue with her children early on using age appropriate language. Another parent expressed that she had simply felt the need to be upfront with her child:

*I talk to my kids about everything…My daughter, when she was four years old she knew about periods, cause it’s a good thing, cause she was nine when she started, so she come running home, I was like ‘oh my gosh’, but she knew exactly what [was happening].* (Mother, Black Parents Focus Group)

### Theme 4: Media as a catalyst and a barrier

Topics such as HIV or STI prevention were not first broached by parents. Rather, the media and the school system tended to be the catalyst that first ignited those discussions. “The TV is inundated with it, and of course our kids all watch Black Entertainment Television (BET) so, it’s all that wrap it up, wrap it up, you know,” a participant from the Black Parents Focus Group said, in reference to BET’s Rap/Wrap It Up public information campaign to address HIV/AIDS and related issues among Black Americans. Parents became involved with the dialogue when children came to them with follow-up questions.

Within the health and education professionals’ focus group a number of participants voiced concern about the social impact of electronic media on parent–child communication in general. Health and education professionals expressed that the increased availability of, and reliance on, technology like cellular phones had had detrimental effects, noting that “parents are working whatever hours or gone all the time and so the actual face-to-face conversation is [reduced]”, and “the technologies that we have now in our society has made it that much easier [not to communication face-to-face]”. Another participant reiterated that children had formed the habit of texting rather than talking. “Now it ain’t conversation, it’s text. Parents and everybody…”

The reliance on multimedia tools for information was cited by one female youth, who specified that the sources she would consult for advice on sexual health varied depending on her location,

*If you’re home then probably it would be the computer, and like books or stuff. But if you’re at school then maybe the health nurse or a certain teacher* (Female, Youth Focus Group)

Another female youth explained that she would, “Google” her query if she was in need of sexual health information.

### Theme 5: Peers as a catalyst

While all participants could recall going to one or more sources to discuss sex and sexual health, a number of participants cited having had discussions with friends that they may not have felt comfortable having with anyone else. In retrospect older participants agreed that the information provided by peers was not always accurate or helpful but peers had served as a sounding board during a time when both their parents and the school system were silent on matters to do with sexuality.

For the younger generation, peers also indirectly served as a catalyst for parent–child dialogue in a number of ways. First, some parents noted that because their children were at times attending class alongside classmates with advanced sexual health knowledge or experience at a younger and younger age, they felt it was important to have a frank discussion with their child in order to safeguard them. Second, one parent noted that her child would sometimes come home and repeat a new piece of information that s/he had learned from a friend. In response, the parent would take the opportunity to discuss the topic with the child to ensure that s/he had the correct information.

### Theme 6: Time constraints as an obstacle

More often than not, participants from the Biracial, Black and Health and Education Professionals Focus Groups voiced concern that Black families were spending less quality time together in large part because of heavy workloads, competing schedules, or unstable home environments. Several of the Black parents explained how they were often too busy working several jobs or long hours at one job, and as a result they had less face-to-face time with their children overall.

*It wears you down when you give so much of yourself [elsewhere] and you don’t have a whole lot left to give to the people who you should be giving it to* (Father, Black Parents Focus Group)

*A lot of parents are single, some of the parents are low income so they’re working two and three jobs, they don’t have that time to talk and other parents are putting their faith into the school system because that’s where the tax dollars are going. The tax dollars are going in the school so they’re expecting the school to pick up where they’re leaving off because they don’t have the time to do so* (Mother, Black Parents Focus Group)

Many participants within the Education and Health Professionals Focus Group discussed how parenting had previously been a community effort with parents looking out for, and stepping in to freely provide guidance and direction to, the children of others in their community. While time constraints may have been present, the community had provided the extra support needed to help the families cope. As one participant recalled, “If we had a party on our street…and one mother came to get her daughter, everybody else’s daughter went, and you couldn’t tell the person, ‘You’re not my mother’. By the time you got home, [your parents] were waiting for you.” They noted a decline in the sense of community within Nova Scotia’s Black community and explained that, “There’s a switch that’s been turned off”. “Nowadays everyone’s [an] individual,” one educator remarked.

## Discussion

The current study is one of the first to examine sexual health communication between Black youth and parents residing in Nova Scotia, Canada. Previous studies supported the use of parents as interveners to help address and reduce risk behaviours which leave youth vulnerable to sexually transmitted infections, yet there was a dearth of research on the sexual health promotion communication experiences of Black families in Canada. Overall, the findings of this study reveal that parent–child communication on sexual health within Black families in Nova Scotia is varied and complex and is heavily affected by the myriad of interpersonal, intrapersonal, structural and environmental determinants of health faced by Black youth and their parents.

Although parents seemed to be aware of the importance of positive parent–child conversations on issues of sex, sexual health, relationships and STIs, our data revealed that most parents generally felt unprepared for, or overwhelmed by, such discussions due in part to their own lack of exposure to such dialogues. As a result, prime opportunities for discussion were sometimes thwarted by communicating in a manner that conveyed disapproval of sexual activity, such as taking away condoms. Children, in turn, react by withholding information about their relationships and sexual activity for fear of upsetting their parents. Therefore, while parents may feel as though their actions have somehow prevented their child’s sexual behaviour, they may have actually unintentionally heightened the risk of negative sexual health outcomes.

With respect to gender as a key determinant of health, the content of discussions varied by the sex of the parent. While most mothers identified themselves as the primary providers of sexual health information, the majority of female and male youth voiced a reluctance to participate in conversations about sex or sexual health with their mothers whose dialogues were often seen as restrictive. Conversations usually centered on the negative consequences of unprotected sex without touching on other topics such as STIs, relationships, sexual development, or positive facets of sexuality, though such knowledge is vital for healthy sexual development in adolescents. Youth frequented other sources for information with greater ease, most notably, fathers, brothers and other male role models or authority figures in the case of male youth, and school nurses, books or the Internet, in the case of female youth. From a health promotion perspective, it is important to note that fathers tended to center their discussions on positive values related to relationship building.

Encouraging open communication with children from an early age increased the ease at which parents could continue conversations about sexual health as their children matured. Given the competing time demands that parents noted, early communication may allow parents to make the best use of what face-to-face time they are able to spend with their children.

The integration of technology into the daily lives of youth both encouraged and hindered communication. Television shows provided a seed for parent–child discussion on matters such as HIV, while the Internet served as a source on sexual health for youth, and reliance on cellular phones interfered with the face-to-face conversations between parents and youth.

Peers were also found to play an indirect role in parent–child communication. Some parents felt compelled to have discussions with their children in order to correct misleading information provided by peers.

The findings from this study have a number of important implications for sexual health promotion programs and interventions that attempt to reduce the burden of STIs and HIV among Black youth. Health promotion interventions and public health programs aimed at reducing both HIV and STIs within Black communities may need to consider fostering parent–child communication by incorporating parents into their intervention strategies. For example, a health promotion campaign (ex: raise awareness about the need for condom use during sex to avoid transmission of a particular STI) might be two pronged; half of the campaign’s key messages could target youth while the other half of the messages could attempt to raise awareness amongst parents about the same issue and encourage parents to foster a dialogue on the issue with their children.

As parents and health and education professionals noted, the decline in community parenting in the face of ongoing structural inequities has only widened the communication gap between Black youth and adults. Trusted community institutions and key individuals within these institutions may serve as critical connectors in helping to prepare parents and youth alike to engage in positive communication. Although the shortcomings of the church in guiding their own sexual health knowledge were noted, parents maintained an optimistic outlook about the role that the church could and should play in the lives of their own children. Many churches remain limited in terms of the type of sexual health and HIV prevention education they can and will deliver, but the creation of church-community health centre partnerships to facilitate periodic discussions on issues of sexuality and sexual health for older youth groups within the church and tailored church messages geared towards encouraging and providing guidance on deeper parent–child communication could be welcomed. Such partnerships with community health centres may allow churches to deliver more evidence-based prevention information that could be of optimal use to youth.

Although schools were not found to provide Black youth with useful sexual health promotion information at the high school level, youth could recall having had a sexual education course at the junior high level. Integration of a component on effective communication into junior high school sexual health education courses could help prepare youth on how best to initiate conversations on sexual health with their peers, partners and parents in later years.

In light of the fact that Black women in Canada continue to be disproportionately affected by HIV/AIDS [34] and Canadian women between the ages of 15–19 are most at risk for HIV [35], our findings serve as a call to action in meeting the sexual health promotion needs of Black Nova Scotian youth. Specifically, the finding that HIV and other STIs were not regular, if ever, topics of discussion between children and their parents is concerning. Innovative sexual health promotion interventions that seek to address the gaps in knowledge surrounding HIV risk among Black youth through, for instance, social media, are urgently needed.

Although this study sheds new light on issues specific to sexual health communication in the context of Black youth and their parents in Nova Scotia, there are several limitations to be noted. The choice of snowball sampling method may have biased the sample in favour of Indigenous Black Nova Scotians and those more willing to discuss the topic of sexual health communication. It is also important to note that the findings of this study are not meant to represent the broad spectrum of views and experiences of all Black Nova Scotians and are therefore not generalizable to the experiences of other Black Canadians.

## Conclusion

Key findings from this study suggest that Black Nova Scotian youth and their parents face a number of key challenges in dialoguing about sex, sexual health, and the prevention of STIs and HIV, in particular. While parents have the potential to play a pivotal role in the sexual health promotion and education of Black youth, they require additional community supports and resources to succeed. Specifically, community institutions such as schools, health centres and churches could serve as important partners, not only by providing sexual health education to youth, but also by encouraging and facilitating opportunities for health promoting parent–child dialogue more broadly.

## Competing interests

The authors confirm that they have no competing interests with regards to the publication of this article.

## Authors’ contributions

AND undertook the qualitative data collection and analysis, and wrote the manuscript. JCG was the principal investigator of the study in Nova Scotia, conceived of the paper, and reviewed and refined the manuscript. CG was a co-principal investigator, conceived the Parent–child Communication study’s development and design, and reviewed the manuscript. All authors have read and approved the final manuscript.
